# Visual Inhibition Measures Predict Speech-in-Noise Perception Only in People With Low Levels of Education

**DOI:** 10.3389/fpsyg.2018.02779

**Published:** 2019-01-23

**Authors:** Sarah Knight, Antje Heinrich

**Affiliations:** ^1^Speech, Hearing & Phonetic Sciences, University College London, London, United Kingdom; ^2^Medical Research Council Institute of Hearing Research, University of Nottingham, Nottingham, United Kingdom; ^3^Manchester Centre for Audiology and Deafness, University of Manchester, Manchester, United Kingdom

**Keywords:** speech-in-noise, inhibition, aging, Stroop tasks, educational attainment

## Abstract

Inhibition—the ability to suppress goal-irrelevant information—is thought to be an important cognitive skill in many situations, including speech-in-noise (SiN) listening. Both inhibition and SiN perception are thought to worsen with age, but attempts to connect age-related declines in these two abilities have produced mixed results even though a clear positive relationship has generally been hypothesized. We suggest that these inconsistencies may occur because listener-based demographic variables such as educational attainment modulate the relationship between inhibition and SiN perception. We tested this hypothesis with a group of 50 older adults (61–86 years, mean: 69.5) with mild-to-moderate age-related hearing loss (8–53 average dB HL, mean: 25.3 dB HL). Participants performed a visual Stroop task and two SiN tasks. In a Stroop task one stimulus dimension is named while a second, more prepotent dimension is ignored. Results show a clear influence of educational attainment on the relationship of visual Stroop scores to SiN performance, but only for those with lower levels of education. These findings highlight for the first time the importance of considering potentially heterogeneous demographic listener variables when analyzing cognitive tasks and their relationship to SiN perception.

## Introduction

Inhibition—the ability to suppress goal-irrelevant information—is thought to be important across a range of tasks (MacLeod, 1991). One such task is speech-in-noise (SiN) perception (Sommers and Danielson, 1999; Janse, 2012), in which listeners must focus on the target speech (foreground) and ignore any distracting sounds (background). Inhibition has been suggested to decline with age (Hasher and Zacks, 1988), with negative implications for a range of cognitive domains and, consequently, everyday activities (Stoltzfus et al., 1996; Burke, 1997). The ability to understand SiN is also observed to worsen with age [Committee on Hearing and Bioacoustics and Biomechanics (CHABA), 1988]. Given the suggested importance of inhibition for SiN perception, researchers have begun to investigate the possibility that age-related declines in inhibition may account, at least in part, for the difficulties older listeners have when listening in noisy environments. However, this task has been complicated by the fact that listener variables, both sensory and cognitive, may moderate the relationship. In a previous paper (Knight and Heinrich, 2017) we investigated the potential for sensory changes—in this case, hearing sensitivity—to affect the relationship between inhibition and SiN perception. Here we look at the moderating effect of a cognitive variable—educational attainment. The influence of educational attainment on the relationship between inhibitory abilities and SiN perception is largely unexplored, despite evidence suggesting that education can affect performance on inhibition tasks across the lifespan (Van der Elst et al., 2006).

### Stroop Tasks

Stroop tasks (Stroop, 1935) are one common means of assessing inhibition. In the traditional color-word Stroop task, participants are asked to name the ink color of a string of letters. The string can be either meaningless (e.g., XXXX; neutral condition), or can form a conflicting color word (e.g., BLUE printed in red; incongruent condition). Word reading is a more prepotent response than color naming, and as a result has the potential to interfere with color naming (Melara and Algom, 2003); participants must therefore attempt to inhibit the conflicting word. The difference in reaction time (RT) between color naming in the neutral and incongruent conditions is taken as measure of inhibitory ability, and is termed Stroop interference (SI).

It has been suggested that the observed age-related changes in visual Stroop performance could in fact be due, at least in part, to additional processes (i.e., non-inhibitory factors), and that scoring methods should be used which account for these processes (Ben-David and Schneider, 2009). In Knight and Heinrich (2017) we detailed the development of scoring methods designed to account for some of these extraneous age-related changes and investigated the influence of these scoring methods on the relationship between inhibition and SiN perception. However, for this particular dataset, the traditional and alternative visual Stroop scores were shown to be closely correlated, suggesting that sensory (color vision) and inhibitory declines occurred in a comparable fashion in this cohort. For this reason, and to maximize comparability with existing studies, we only report results involving the traditional Stroop scoring method here. In Knight and Heinrich (2017), we also reported results from an auditory version of the traditional Stroop task. However, as discussed in that paper, this task did not produce a robust Stroop effect, and its relationship to both the visual Stroop and SiN tasks was unclear. For this reason, we do not examine results involving the auditory Stroop task here.

### Inhibition and SiN

Two potential ways in which inhibition might affect SiN perception have been proposed. First, it has been suggested that poor inhibition may affect SiN perception by increasing susceptibility to background noise (Janse, 2012). Second, it has been suggested that poor inhibition may make it harder for the listener to successfully select the target during lexical access (Sommers and Danielson, 1999).

#### Lexical Access

The Neighborhood Activation Model (NAM) proposes that items in the mental lexicon are organized into similarity neighborhoods, defined as all words that can be created from a target item by adding, deleting or substituting a single phoneme (Luce and Pisoni, 1998). A stimulus word will activate both the target word and, to varying degrees, its neighborhood. For any given word, the neighborhood may be small (sparse) or large (dense); additionally, words will be activated more strongly if they are more commonly encountered (high frequency of occurrence) than if they are relatively rare (low frequency of occurrence). High-frequency words with a sparse neighborhood as therefore classed as “lexically easy,” and low-frequency words with a dense neighborhood as “lexically hard” (e.g., Taler et al., 2010). Lexically easy words are more easily recognized than lexically hard words (Sommers and Danielson, 1999; Taler et al., 2010; Helfer and Jesse, 2015). Inhibition is thought to be more important in the perception of lexically hard words than lexically easier words (Sommers and Danielson, 1999; Taler et al., 2010).

Semantic context can also affect lexical access by increasing the likelihood that a given word will occur. Recognition is better for words in semantically meaningful sentences than words in isolation (Miller et al., 1951; Nittrouer and Boothroyd, 1990), and for words within sentences that are more, as opposed to less, predictable (Bilger et al., 1984).

#### Inhibition and Lexical Access

Older adults may be a particularly informative participant group to examine with respect to the relationship between lexical access and inhibition, as age-related changes in the effects of lexical difficulty and semantic context have been taken as evidence for age-related declines in inhibitory ability. For example, older adults appear to be disproportionately poor at identifying words with dense neighborhoods compared to younger adults, in both the auditory and audio-visual domains (Sommers, 1996; Dey and Sommers, 2015). Since competing words from the target neighborhood have to be suppressed for successful word identification, this has been interpreted as reflecting an age-related decline in inhibitory abilities. Similarly, older listeners appear to benefit more than younger listeners from the addition of semantic context (Pichora-Fuller et al., 1995), possibly because of increased linguistic experience (Sommers and Danielson, 1999). However, the relationship between SiN perception and inhibition—as measured by traditional tasks such as Stroop tasks—is not clear-cut. Several studies fail to show any kind of relationship (Gilbert et al., 2013; Helfer and Freyman, 2014). In our dataset, we predicted a negative relationship between Stroop scores and SiN perception (with a lower—and therefore better—Stroop score suggesting a higher—and therefore better—score on the SiN task); however, we found this relationship only for certain groups of listeners performing certain types of SiN task (Knight and Heinrich, 2017).

#### Conflicting Findings Regarding Inhibition and SiN

Conflicting findings regarding the importance of inhibition for SiN perception may be due to a number of factors. First, methodological issues—such as the use of Stroop tasks in different modalities (auditory vs. visual) and/or the use of scoring methods which either do or do not account for possible confounding factors (Knight and Heinrich, 2017)—may lead to inconsistent results. It is also possible that, in some cases, the between-subject variability arising from standard inhibition tasks is too low to allow for correlational studies of individual differences (Hedge et al., 2018). Second, it seems likely that there is an impact of listener variables on the relationship between Stroop scores and SiN perception. In the current study, the two important listener variables were sensory (perceptual) and cognitive abilities. With regard to sensory abilities, we found only a limited influence of hearing sensitivity on the Stroop/SiN relationship (Knight and Heinrich, 2017). However, our auditory stimuli were presented at 30 dB above each listener's individual speech reception threshold (SRT), which may have mitigated to some extent difficulties caused by poorer hearing sensitivity. The role of hearing sensitivity might also reasonably be expected to be small when using non-auditory (i.e., visual) Stroop tasks. With regard to cognitive abilities, we know that SiN perception is thought to be influenced by cognition (Akeroyd, 2008), and inhibition is generally suggested to be a core cognitive (as opposed to sensory) ability (Conway and Engle, 1994; Friedman and Miyake, 2004; Baddeley, 2012; Diamond, 2013). It therefore seems likely that any listener factors which affect the cognition variable under consideration (in this case, inhibition) will also have a significant influence on the Stroop/SiN relationship.

### The Role of Educational Attainment

Van der Elst et al. (2006) found that older adults with low levels of educational attainment performed worse than those with average and high levels of educational attainment on a Stroop color-word task. Specifically, the authors reported significantly larger Stroop interference scores for the low education group (i.e., a larger difference between the interference condition and the neutral/reading conditions). This general finding has subsequently been reproduced in several studies across different languages and cultures, although the exact nature of the Stroop tasks used and the method of calculating the scores varies (e.g., Zalonis et al., 2009; Rivera et al., 2015).

This finding is not limited to Stroop effects: educational attainment has been shown to correlate with older adults' scores on a range of cognitive tasks (Evans et al., 1993; Gallacher et al., 1999). In all cases, higher educational attainment was connected to better performance. Educational attainment has been proposed to contribute to cognitive reserve (Stern, 2002), and indeed is assumed to be an indirect measure of cognitive reserve (Van Dijk et al., 2008). Cognitive reserve refers to the extent to which individuals can maintain high levels of cognitive performance despite brain pathology or cognitive decline. It is suggested to be related to physical capacity, such as neuronal network density, the efficient use of cognitive resources, and/or the recruitment of alternative processing strategies.

Educational attainment may also have some “protective” effect, mitigating age-related cognitive decline to some extent (Stern, 2002) although this claim is contested (Anstey and Christensen, 2000; Meijer et al., 2009). Nevertheless, even studies which show no protective effect of education on cognitive decline over time still show an overall enhanced performance on cognitive tasks, including Stroop color-word tasks, for those with higher levels of educational attainment (Van Dijk et al., 2008).

Since educational attainment appears to influence performance on color-word Stroop tasks, and possibly other types of Stroop task too, we expect participants with higher levels of educational attainment to perform better (i.e., show a smaller Stroop interference score) on the Stroop task we used. The influence of educational attainment on SiN tasks is less well-documented, but there is some evidence to suggest that those with lower levels of education perform worse on word-learning tasks in the presence of irrelevant speech (Meijer et al., 2006). If educational attainment significantly modifies performance on Stroop tasks, but does not affect performance on a speech-in-noise task—or affects it along different lines—then meaningful relationships between SI scores and SiN perception may be obscured if education is not taken into account. Specifically, if higher levels of education allow participants to compensate to some extent for declines in inhibitory ability, then the relationship between Stroop scores and SiN perception may not be observed as strongly for those with high educational attainment. Further to this, the relationship between Stroop scores and SiN perception should be particularly strong when the SiN stimuli demand high levels of inhibition—that is, at lower (less favorable) SNRs, when sentential context is lacking (i.e., when targets are isolated words), when target words have a low word frequency and/or high neighborhood density, or when semantic context does not aid inference (i.e., when targets appear in low-predictability sentences). As a result, differences in the Stroop/SiN relationship between those with higher and lower levels of education may be most apparent in these conditions.

### The Current Study

In this paper, we carry out an exploratory analysis to assess the degree to which Stroop interference scores are sensitive to a simple measure of education, and the ways in which educational attainment affects the relationship of Stroop scores to speech-in-noise perception in a group of older adults with a wide range of ages. Basic performance on the SiN and Stroop tasks, and the relationships between the two, have been reported elsewhere (Knight and Heinrich, 2017). In this previous study, the results were mixed, with Stroop tasks showing different relationships with different types of SiN task. In the current study, using the same dataset, we explore the possibility that by including educational attainment as an additional variable we may clarify some of these earlier results.

## Hypotheses

H1: It is expected that Stroop interference scores will be more strongly related to SiN perception in those with lower educational attainment. If higher levels of education allow participants to compensate to some extent for declines in inhibition, then Stroop interference scores will not be an accurate reflection of inhibitory abilities for these participants. As a result, the relationship between Stroop scores and SiN perception may not be observed as strongly for those with high educational attainment.

H2: Any difference in the relationship between Stroop interference scores and SiN perception between those with higher and lower educational attainment is expected to be more pronounced in listening conditions that demand high levels of inhibition (at lower SNRs, when targets are isolated words, when target words have a low word frequency and/or high neighborhood density, or when targets appear in low-predictability sentences).

## Method

### Participants

The participants were 50 adults aged 60 or older (mean: 69.5 years, SD: 6.4, range = 61–86) with mild hearing loss. A sample size of *N* = 50 allowed detection of a medium-sized effect for the link between Stroop scores and SiN perception (*r* = 0.35) at alpha (two-tailed) = 0.05 with a probability of 80%. This was based on relevant earlier studies (Sommers and Danielson, 1999; Janse, 2012) which typically show medium-to-large effect sizes. Participants were excluded if they reported hearing aid use and/or non-native English language status.

A Landolt C Chart was used to assess visual accuracy, and the City University Color Vision Test was used to assess color vision. All participants were able to read a full line of optotypes on the Landolt C Chart at a logMAR value of at least 0.3, with the majority (34) able to read a full line at between −0.1 and 0.1 logMAR. Four participants failed the Color Vision Test, and the same group also verbally reported color blindness; these participants were excluded. No other participant reported difficulty in reading the test materials for the visual Stroop task. All participants were screened for mild cognitive impairment (MCI) using the Montreal Cognitive Assessment (MoCA) (mean: 27.86; SD: 1.95).

The results reported in this paper form part of a larger study into cognitive contributions to speech perception in older adults. Unreported results do not relate to the topics discussed here. The study was carried out in accordance with the recommendations of the University of Nottingham's Code of Research Conduct and Research Ethics, with written informed consent from all participants. All participants gave written informed consent in accordance with the Declaration of Helsinki. The protocol was approved by the University of Nottingham's School of Psychology Ethics Committee (ref. 464).

### Auditory Measures

**Figure 1:**
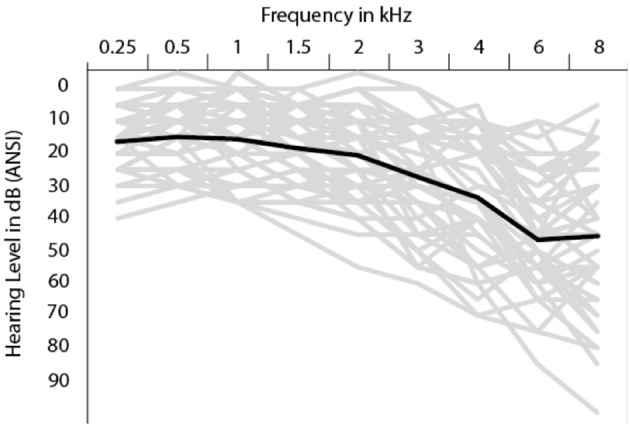
Mean PTA thresholds (black line) and individual PTA thresholds (gray lines) as a function of frequency.

Pure-tone air-conduction thresholds (PTA) were collected for 9 frequencies between 0.25 and 8 kHz for each ear, following the procedure recommended by the British Society of Audiology (2011) using an Interacoustics Audiometer AT235 (Interacoustics, Middelfart, Denmark) and TDH39P headphones (Telephonics, Farmingdale, NY, USA). Mean thresholds as a function of frequency are presented in Figure 1. As this figure shows, hearing sensitivity varied considerably between participants, particularly at the higher frequencies.

Speech reception thresholds (SRT) were obtained for the left ear only using 30 sentences from the Adaptive Sentence List (MacLeod and Summerfield, 1990). Sentences were initially presented at 60 dB SPL, with a one-down-one-up procedure and step sizes of 10 dB down, then 5 dB up for the first reversal; the remainder of the trials used a three-down-one-up procedure with a step size of 2 dB. The last two reversals were averaged to determine the 79% accuracy point (Levitt, 1971). Based on this, all auditory stimuli used throughout the study were presented to the left ear at 30 dB SL—that is, 30 dB above each participant's individual threshold. This procedure was used to partially control for differences in intelligibility in quiet due to the considerable range in participants' hearing sensitivity.

### Stroop Task

Each participant performed a visual Stroop task. This task was a variant of the traditional color-word Stroop task, modeled after Janse (2012). Participants saw grids of 48 boxes, presented in an 8 x 6 arrangement. There were two versions of each of three types of grid: (i) a reading grid, consisting of white boxes containing black color words; (ii) a control grid, consisting of colored boxes containing the string “XXXX” in black; (iii) an interference grid, consisting of colored boxes containing mismatched color words in black. For (i), the task was to read the words aloud as quickly and accurately as possible. For (ii) and (iii), the task was to name the background color of the boxes as quickly and accurately as possible. The total time to complete each grid was timed by the experimenter using a stopwatch, and overall time for each grid type obtained by averaging the times from the two versions. A traditional SI measure was obtained by subtracting overall time in the control condition from overall time in the interference condition. Mean performance per item was 0.32 s (SD: 0.09 s; range: 0.14–0.54 s). Participants occasionally made errors on the interference grid. These were not penalized if the participant corrected their mistake without prompting. Uncorrected mistakes were penalized by calculating the participant's average time per item on the relevant interference grid, then adding this duration to the total grid time for each uncorrected mistake. The times for the reading and control grids represented error-free performance for all participants.

### Speech-in-Noise Tasks

SiN tasks varied in both semantic predictability and lexical difficulty. In the sentence task, targets were the final words of low-predictability (LP) and high-predictability (HP) sentences, where predictability was varied by altering the preceding context. The sentences used were from a recently developed sentence pairs test (Heinrich et al., unpublished) based on the SPIN-R test (Bilger et al., 1984). In the word task, targets were isolated words whose lexical difficulty was varied in terms of word frequency (WF) and neighborhood density (ND). Lexical information (word frequency/neighborhood density) for the target words is presented in Table 1.

**Table 1:** Lexical information for word stimuli.

		**LOW WF LOW ND**	**LOW WF HIGH ND**	**HIGH WF LOW ND**	**HIGH WF HIGH ND**
WF	Max	9,879	8,958	41,358	62,803
	Min	106	117	10,152	10,029
ND	Max	18	38	18	35
	Min	2	19	2	19

All stimuli were spoken by a male Standard British English speaker and presented in speech-modulated noise (SMN) derived from the sentences. Two SNRs were used to create a more or less adverse listening condition (words at +1 and −2 dB; sentences at −4 and −7 dB). SNR levels were chosen to vary the overall difficulty of the task between 20 and 80 percent accuracy.

After hearing each sentence or word participants repeated as much as they could. Testing was self-paced, and responses were recorded for offline scoring.

### Educational Attainment

As a basic measure of educational attainment, participants were asked to specify the number of years they had spent in education. In general, 10 years of education corresponded to leaving school at age 16, 12 years to leaving school at age 18, and each year of higher education (university, training college etc.) was added as required. The mean number of years in education in our participant group was 14 (range: 5–22). Poorer hearing in adults is sometimes found to be linked to lower educational attainment (e.g., Cruickshanks et al., 2010). However, in our data, the correlation between the two (with both measures centered) was only small and not significant (*r* = −0.264, *p* = 0.064).

### Modeling

The outcome measure was speech intelligibility, transformed into RAUs (Studebaker, 1985). Five stimulus-based variables were coded as categorical predictors: (i) semantic predictability (“Pred”; LP/HP) of sentence-final words; (ii) word frequency (“WF”; high/low) and (iii) neighborhood density (“ND”; high/low) of isolated words; (iv) speech type (“Type”; sentences/words); (v) SNR (“SNR”; high/low).

Three listener variables were coded as continuous predictors: (i) Stroop interference score (“SI”); (ii) hearing sensitivity (“PTA”); (iii) educational attainment (“Edu”). The PTA variable was calculated by averaging the obtained thresholds at all tested frequencies in the left ear for each participant, and then centering these values. The educational attainment variable was calculated by centering each participant's reported years of education. We decided to center these variables to facilitate comparison of results across studies and cohorts.

The relationship between these predictors and the outcome measure was assessed using a series of linear mixed models (LMMs) using ML estimation, with predictor variables as fixed effects and Type 3 SS. All models included participants as random intercepts. Given that smaller Stroop interference scores reflect better performance, we expected negative relationships between Stroop scores and SiN performance as a general rule. A backwards stepwise procedure was used to determine the final set of predictors for each model. This procedure was implemented through manual checking and effect removal. Full details of this procedure and of the models run for each step are documented in Appendix 1.

For the final set of models, plots were used to visually assess whether assumptions of normality of residuals had been violated. For each model, three plots were generated: (i) QQ plot of model residuals; (ii) QQ plot of random subject effects; (iii) plot of residuals vs. predicted values. These plots are given in Appendix 2. The plots suggest that assumptions were generally not violated.

All analyses were performed in R (version 3.5.1) using the packages lmerTest (to run models and significance tests), MuMln (to obtain R2 values) and HLMdiag (to assess model assumptions).

## Results

For details of the effects of stimulus-based variables (speech type, predictability, WF, ND) and main effects of Stroop, please see Knight and Heinrich (2017). In this paper, we focus on the interaction of educational attainment with these variables, and particularly the influence of educational attainment on the relationship between Stroop scores and SiN perception.

The correlation between Stroop scores and educational attainment overall was −0.184 (*p* > 0.1).

The outcome measure—SiN perception—was explored for high- and low-predictability sentences and for single words varying in word frequency and neighborhood density. Three separate analyses were run in order to investigate the effect of the predictor variables for the sentence task, the word task and the sentence and word task combined. The analyses combining the scores from the sentence and word tasks was included in order to directly compare the effect of Stroop scores and their interaction with educational attainment across the two outcome measures.

Tables 2–4 indicate, for each dataset, the final model predictors, fixed effect estimates, and *F*-tests (with *p*-values) indicating the significance of each term. Interactions which include the Stroop measure but not the education variable are listed in the tables but not discussed (please see Knight and Heinrich, 2017 for discussion of interactions involving only Stroop measures and/or stimulus-based variables and PTA).

**Table 2:** Summary of LMM assessing influence of educational attainment on the relationship of visual Stroop scores to sentence perception.

**DATASET: Sentences**			
**Model summary**			
AIC = 1391.09 BIC = 1420.02 R2 (marginal) = 0.79 R2 (conditional) = 0.84			
**ANOVA (Type III, Satterthwaite's method)**			
**Fixed effects**	**Fixed-effect estimates**	***F*****-value (NumDF, DenDF)**	***p*****-value**
(Intercept)	91.72		
SI	−3.69	0.10 (1, 46)	0.754
PTA	−0.65	40.03 (1, 46)	<0.001^***^
Edu	−2.20	5.03 (1, 46)	0.030^*^
Pred	−32.66	597.71 (1, 138)	<0.001^***^
SNR	−15.84	140.57 (1, 138)	<0.001^***^
SI*Edu	**7.81**	**7.39 (1, 46)**	0.009^**^

*** < 0.001;

** < 0.01;

* *< 0.05*.

*Bold text indicates an interaction between Stroop scores and educational attainment*.

**Table 3:** Summary of LMM assessing influence of educational attainment on the relationship of visual Stroop scores to word perception.

**DATASET: Words**			
**Model summary**			
AIC = 2690.22 BIC = 2748.84 R2 (marginal) = 0.48 R2 (conditional) = 0.72			
**ANOVA (Type III, Satterthwaite's method)**			
**Fixed effects**	**Fixed-effect estimates**	***F*****-value (NumDF, DenDF)**	***p*****-value**
(Intercept)	70.65		
SI	−4.87	1.27 (1, 46)	0.27
PTA	−0.46	12.64 (1, 46)	<0.001^***^
Edu	0.31	1.24 (1, 46)	0.27
SNR	−8.15	81.95 (1, 322)	<0.001^***^
WF	3.63	60.07 (1, 322)	<0.001^***^
ND	20.98	13.47 (1, 322)	<0.001^***^
SI^*^ND	−19.75	4.90 (1, 322)	0.028^*^
PTA^*^Edu	0.01	1.18 (1, 46)	0.283
PTA^*^SNR	0.04	0.17 (1, 322)	0.682
Edu^*^SNR	0.23	0.73 (1, 322)	0.394
WF^*^ND	−20.03	147.78 (1, 322)	<0.001^***^
PTA^*^Edu^*^SNR	0.06	5.12 (1, 322)	0.024^*^

*** < 0.001;

** < 0.01;

* *< 0.05*.

**Table 4:** Summary of LMM assessing influence of educational attainment on the relationship of visual Stroop scores to the combined dataset.

**DATASET: Combined**			
**Model summary**			
AIC = 1233.10 BIC = 1271.68 R2 (marginal) = 0.61 R2 (conditional) = 0.84			
**ANOVA (Type III, Satterthwaite's method)**			
**Fixed effects**	**Fixed-effect estimates**	***F*****-value (NumDF, DenDF)**	***p*****-value**
(Intercept)	73.85		
SI	−4.14	1.64 (1, 46)	0.207
PTA	−0.61	32.14 (1, 46)	<0.001^***^
Edu	−1.76	3.73 (1, 46)	0.060
Type	7.34	13.93 (1, 138)	<0.001^***^
SNR	−14.89	251.58 (1, 138)	<0.001^***^
SI^*^Edu	**6.67**	**6.23 (1, 46)**	0.016^*^
SI^*^Type	−19.50	5.65 (1, 138)	0.019^*^
PTA^*^Type	0.14	3.98 (1, 138)	0.048^*^
Type^*^SNR	5.82	14.85(1, 138)	<0.001^***^

*** < 0.001;

** < 0.01;

* *< 0.05*.

*Bold text indicates an interaction between Stroop scores and educational attainment*.

As Tables 2 and 4 indicate, a significant two-way interaction of SI x Edu is observed for both the sentence task and the combined SiN data (marked in bold). This interaction indicates that the slope relating SiN performance to Stroop interference is negative for those with low education and positive for those with high education. In other words, the predicted negative relationship between Stroop scores and SiN perception was only seen for those with relatively low levels of education. For those with relatively high levels of education, the relationship was typically positive (i.e., in the unexpected direction)^1^. For the word task, no interaction between educational attainment and Stroop scores was observed (Table 3).

## Discussion

Inhibition is a core cognitive ability which has been suggested to be important for speech-in-noise (SiN) perception. However, attempts to establish the link between inhibition and performance on SiN tasks may have been complicated, particularly among older adults, by the influence of listener variables such as hearing sensitivity and educational attainment. Here we set out to examine the influence of educational attainment on the relationship between Stroop scores and SiN performance. In all cases, the result of interest was a potential modification of the relationship between Stroop scores and SiN perception scores by educational attainment, indicating an influence of educational attainment on the way in which Stroop scores relate to performance on a set of speech-in-noise tasks.

We used Stroop interference (SI) scores derived from a version of the classic color-word Stroop task. The SiN tasks were designed to probe different ways in which inhibition might be important for speech perception. First, all tasks were presented in noise, since inhibition is suggested to be important in reducing susceptibility to background noise (Janse, 2012). Second, target speech varied either in lexical difficulty (word frequency and neighborhood density of isolated words) or semantic context (sentences), since these characteristics have been suggested to place different demands on inhibition (Sommers and Danielson, 1999). For each participant, we also obtained measures of educational attainment (years of education) and hearing sensitivity (an average of PTA thresholds across frequencies from 0.25 to 8 kHz).

### Educational Attainment and Stroop Tasks

We found no evidence that participants with higher levels of educational attainment performed better (i.e., showed a smaller Stroop interference score) on the Stroop task: there was no significant correlation between Stroop scores and educational attainment overall. This is in contrast to results from a number of other studies which suggest that educational attainment boosts performance on Stroop tasks (Van der Elst et al., 2006; Zalonis et al., 2009; Rivera et al., 2015) and on cognitive tasks more generally (Evans et al., 1993; Gallacher et al., 1999). This discrepancy could be due to our rather small and homogenous sample of participants: the participants were self-selecting and as such were likely to have included older adults with high levels of interest in academic research and general intellectual engagement, even if their formal education was relatively brief. Other studies, by contrast, used relatively large sample sizes of heterogeneous participants, seeking population norms.

### Influence of Education on the Stroop/SiN Relationship

*H1: It is expected that Stroop interference scores will be more strongly related to SiN perception in those with lower educational attainment*.

In general, we expected to see a negative Stroop/SiN relationship, with larger Stroop effects corresponding to lower scores (i.e., worse performance) on SiN tasks; we also expected this relationship to be stronger for those with lower educational attainment. This hypothesis was supported. Indeed, the predicted negative Stroop/SiN relationship was observed *only* for those with lower educational attainment. Such an influence of educational attainment has the potential to be very important for studies attempting to link cognitive abilities to SiN performance in groups with heterogeneous educational histories, and may have broader implications for understanding how and when traditional cognitive measures relate to other abilities (such as SiN perception) in the population as a whole. However, as discussed further below, this effect must be replicated in larger studies with more diverse subject pools before any firm conclusions can be drawn about its validity or underlying mechanisms.

It is also worth noting that the interaction between Stroop interference scores and educational attainment was observed for the sentence-based SiN task but not for the SiN task involving isolated words, and it seems likely that the same interaction in the combined dataset was driven by the results from the sentence task. One might therefore expect the three-way interaction of SI x Edu x Type to have been significant in the combined dataset, but it was not; this may be due to insufficient power. Further work is needed to better understand the interaction of inhibitory abilities and educational attainment during SiN listening involving different types of linguistic information.

*H2: Any difference in the relationship between Stroop interference scores and SiN perception between those with higher and lower educational attainment is expected to be more pronounced in listening conditions that demand high levels of inhibition*.

This hypothesis was not supported. Any influence of education on the Stroop/SiN relationship took the form of a basic two-way interaction between Stroop scores and educational attainment, with no influence of stimulus-based variables such as SNR, lexical difficulty or semantic context. Interactions of Stroop interference scores with stimulus-based variables are nevertheless present (see Knight and Heinrich, 2017)—but they do not involve educational attainment. This suggests that, whatever variation in cognitive abilities, Stroop response strategies or SiN listening strategies is reflected by the different Stroop/SiN relationships for those with high as opposed to low levels of educational attainment, it does not affect performance on different SiN conditions differently for the two groups.

## Conclusion

In this study we assessed the influence of educational attainment on the relationship between scores from a visual Stroop task and speech-in-noise perception. We used two different speech-in-noise tasks; we also accounted for hearing sensitivity. The results show that educational attainment significantly influenced the relationship between Stroop scores and speech-in-noise (SiN) perception, with the hypothesized relationship observed only for listeners with low educational attainment. The results highlight the importance of considering listener variables that may affect cognitive abilities—such as educational attainment—when attempting to analyse data from cognitive tasks and/or explore their relationship to speech perception. The fact that we observed a relationship between visual Stroop scores and SiN perception suggests that the visual Stroop task may be tapping a modality-independent, general cognitive ability—at least for participants with lower levels of educational attainment.

As discussed in Knight and Heinrich (2017), important limitations of this study include the relatively restricted participant pool and the choice of background masker (non-linguistic speech-modulated noise). Our measure of educational attainment was also somewhat crude: we asked participants to give the number of years they had spent in formal education, but it is possible that their cognitive reserve had been boosted through other activities, such as unreported adult education classes or hobbies. However, other studies also used relatively simple measures of educational attainment; indeed, in many studies education is treated as a categorical variable with only two or three levels, reflecting either different levels of qualification or different durations of formal education. Hence, our measure is typical of the existing literature examining the influence of educational attainment on Stroop scores (e.g., Van der Elst et al., 2006; Zalonis et al., 2009; Rivera et al., 2015). Future work should seek to determine the most useful granularity at which to measure educational attainment, and to attempt to understand more about how and why educational attainment might affect not just performance on cognitive tasks but also the relationship between these tasks and performance on real-world-like tasks such as speech-in-noise perception.

## Author Contributions

AH and SK designed the study and collected the data. SK analyzed the data. AH and SK interpreted the data. SK drafted, and AH contributed to, the manuscript and both contributed to the critical discussions. Both authors approved the final version of the manuscript for publication.

### Conflict of Interest Statement

The authors declare that the research was conducted in the absence of any commercial or financial relationships that could be construed as a potential conflict of interest.

## Footnotes

^1^We describe this interaction as a rate of change where a positive slope indicates an average increase in SiN performance with every additional increase in Stroop interference, while a negative slope indicates an average decrease in SiN performance with every additional increase in Stroop interference. Listener-based variables (Edu and PTA) were always entered as continuous predictors, but we use a categorical median split of Edu in our discussion because it allows for a clearer description of the interaction.

## References

[B1] Akeroyd M. A. (2008). Are individual differences in speech reception related to individual differences in cognitive ability? A survey of twenty experimental studies with normal and hearing-impaired adults. Int. J. Audiol. 47, S53–S71. 10.1080/14992020802301142 19012113

[B2] Anstey K. Christensen H. (2000). Education, activity, health, blood pressure and apolipoprotein E as predictors of cognitive change in old age: a review. Gerontology 46, 163–177. 10.1159/000022153 10754375

[B3] Baddeley A. (2012). Working memory: theories, models, and controversies. Annu. Rev. Psychol. 63, 1–29. 10.1146/annurev-psych-120710-100422 21961947

[B4] Ben-David B. M. Schneider B. A. (2009). A sensory origin for color-word Stroop effects in aging: a meta-analysis. Aging Neuropsychol. Cogn. 16, 505–534. 10.1080/13825580902855862 19479479

[B5] Bilger R. C. Nuetzel J. M. Rabinowitz W. M. Rzeczkowski C. (1984). Standardization of a test of speech perception in noise. J. Speech Lang. Hear. Res. 27, 32–48. 10.1044/jshr.2701.32 6717005

[B6] British Society of Audiology (2011). Recommended Procedure for the Pure Tone Air and Bone Conduction Threshold Audiometry with and without the Use of Masking and Determination of Uncomfortable Loudness Levels. Available online at: http://www.thebsa.org.uk/wp-content/uploads/2014/04/BSA_RP_PTA_FINAL_24Sept11_MinorAmend06Feb12.pdf

[B7] Burke D. M. (1997). Language, aging, and inhibitory deficits: evaluation of a theory. J. Gerontol. Ser. B Psychol. Sci. Soc. Sci. 52, P254–P264. 10.1093/geronb/52B.6.P254 9403514

[B8] Committee on Hearing and Bioacoustics and Biomechanics (CHABA) (1988). Speech understanding and aging. J. Acoust. Soc. Am. 83, 859-895. 10.1121/1.395965 3281988

[B9] Conway A. R. Engle R. W. (1994). Working memory and retrieval: a resource-dependent inhibition model. J. Exp. Psychol. Gen. 123, 354–373. 10.1037/0096-3445.123.4.354 7996121

[B10] Cruickshanks K. J. Nondahl D. M. Tweed T. S. Wiley T. L. Klein B. E. Klein R.. (2010). Education, occupation, noise exposure history and the 10-yr cumulative incidence of hearing impairment in older adults. Hear. Res. 264, 3–9. 10.1016/j.heares.2009.10.008 19853647 PMC2868082

[B11] Dey A. Sommers M. S. (2015). Age-related differences in inhibitory control predict audiovisual speech perception. Psychol. Aging 30, 634–646. 10.1037/pag0000033 26121287 PMC5757834

[B12] Diamond A. (2013). Executive functions. Annu. Rev. Psychol. 64, 135–168. 10.1146/annurev-psych-113011-143750 23020641 PMC4084861

[B13] Evans D. A. Beckett L. A. Albert M. S. Hebert L. E. Scherr P. A. Funkenstein H. H.. (1993). Level of education and change in cognitive function in a community population of older persons. Ann. Epidemiol. 3, 71–77. 10.1016/1047-2797(93)90012-S 8287159

[B14] Friedman N. P. Miyake A. (2004). The relations among inhibition and interference control functions: a latent-variable analysis. J. Exp. Psychol. Gen. 133, 101. 10.1037/0096-3445.133.1.101 14979754

[B15] Gallacher J. E. Elwood P. C. Hopkinson C. Rabbitt P. M. Stollery B. T. Sweetnam P. M.. (1999). Cognitive function in the Caerphilly study: associations with age, social class, education and mood. Eur. J. Epidemiol. 15, 161–169. 10.1023/A:1007576324313 10204646

[B16] Gilbert J. L. Tamati T. N. Pisoni D. B. (2013). Development, reliability, and validity of PRESTO: a new high-variability sentence recognition test. J. Am. Acad. Audiol. 24, 26–36. 10.3766/jaaa.24.1.4 23231814 PMC3683852

[B17] Hasher L. Zacks R. T. (1988). Working memory, comprehension, and aging: a review and a new view. Psychol. Learn. Motiv. 22, 193–225. 10.1016/S0079-7421(08)60041-9

[B18] Hedge C. Powell G. Sumner P. (2018). The reliability paradox: why robust cognitive tasks do not produce reliable individual differences. Behav. Res. Methods 50, 1166–1186. 10.3758/s13428-017-0935-1 28726177 PMC5990556

[B19] Heinrich A. Knight S. (2016). The contribution of auditory and cognitive factors to intelligibility of words and sentences in noise, in Physiology, Psychoacoustics and Cognition in Normal and Impaired Hearing, Vol. 894, eds van Dijk P. Baskent D. Gaudrain E. de Kleine E. Wagner A. Lanting C. (Bonn: Springer), 37–45. 10.1007/978-3-319-25474-6_5 27080644

[B20] Helfer K. S. Freyman R. L. (2014). Stimulus and listener factors affecting age-related changes in competing speech perception. J. Acoust. Soc. Am. 136, 748–759. 10.1121/1.4887463 25096109 PMC4187459

[B21] Helfer K. S. Jesse A. (2015). Lexical influences on competing speech perception in younger, middle-aged, and older adults. J. Acoust. Soc. Am. 138, 363–376. 10.1121/1.4923155 26233036 PMC4506307

[B22] Janse E. (2012). A non-auditory measure of interference predicts distraction by competing speech in older adults. Aging Neuropsychol. Cogn. 19, 741–758. 10.1080/13825585.2011.652590 22293017

[B23] Knight S. Heinrich A. (2017). Different measures of auditory and visual Stroop interference and their relationship to speech intelligibility in noise. Front. Psychol. 8:230. 10.3389/fpsyg.2017.00230 28367129 PMC5355492

[B24] Levitt H. (1971). Transformed up-down methods in psychoacoustics. J. Acoust. Soc. Am. 49, 467–477. 10.1121/1.1912375 5541744

[B25] Luce P. A. Pisoni D. B. (1998). Recognizing spoken words: the neighborhood activation model. Ear Hear. 19, 1–36. 10.1097/00003446-199802000-00001 9504270 PMC3467695

[B26] MacLeod A. Summerfield Q. (1990). A procedure for measuring auditory and audiovisual speech-reception thresholds for sentences in noise: rationale, evaluation, and recommendations for use. Br. J. Audiol. 24, 29–43. 10.3109/03005369009077840 2317599

[B27] MacLeod C. M. (1991). Half a century of research on the Stroop effect: an integrative review. Psychol. Bull. 109, 163–203. 10.1037/0033-2909.109.2.163 2034749

[B28] Meijer W. A. De Groot R. H. Van Boxtel M. P. Van Gerven P. W. Jolles J. (2006). Verbal learning and aging: combined effects of irrelevant speech, interstimulus interval, and education. J. Gerontol. Ser. B Psychol. Sci. Soc. Sci. 61, P285–P294. 10.1093/geronb/61.5.P285 16960232

[B29] Meijer W. A. van Boxtel M. P. Van Gerven P. W. van Hooren S. A. Jolles J. (2009). Interaction effects of education and health status on cognitive change: a 6-year follow-up of the Maastricht Aging Study. Aging Ment. Health 13, 521–529. 10.1080/13607860902860821 19629776

[B30] Melara R. D. Algom D. (2003). Driven by information: a tectonic theory of Stroop effects. Psychol. Rev. 110, 422–471. 10.1037/0033-295X.110.3.422 12885110

[B31] Miller G. A. Heise G. A. Lichten W. (1951). The intelligibility of speech as a function of the context of the test materials. J. Exp. Psychol. 41, 329–335. 10.1037/h0062491 14861384

[B32] Nittrouer S. Boothroyd A. (1990). Context effects in phoneme and word recognition by young children and older adults. J. Acoust. Soc. Am. 87, 2705–2715. 10.1121/1.399061 2373804

[B33] Pichora-Fuller M. K. Schneider B. A. Daneman M. (1995). How young and old adults listen to and remember speech in noise. J. Acoust. Soc. Am. 97, 593–608. 10.1121/1.412282 7860836

[B34] Rivera D. Perrin P. B. Stevens L. F. Garza M. T. Weil C. Saracho C. P.. (2015). Stroop color-word interference test: normative data for the Latin American Spanish speaking adult population. NeuroRehabilitation 37, 591-−624. 10.3233/NRE-151281 26639926

[B35] Sommers M. S. (1996). The structural organization of the mental lexicon and its contribution to age-related declines in spoken-word recognition. Psychol. Aging 11, 333–341. 10.1037/0882-7974.11.2.333 8795062

[B36] Sommers M. S. Danielson S. M. (1999). Inhibitory processes and spoken word recognition in young and older adults: the interaction of lexical competition and semantic context. Psychol. Aging 14, 458–472. 10.1037/0882-7974.14.3.458 10509700

[B37] Stern Y. (2002). What is cognitive reserve? Theory and research application of the reserve concept. J. Int. Neuropsychol. Soc. 8, 448–460. 10.1017/S1355617702813248 11939702

[B38] Stoltzfus E. R. Hasher L. Zacks R. T. (1996). Working memory and aging: current status of the inhibitory view, in Working Memory and Human Cognition, eds Richardson J. T. E. Engle R. W. Hasher L. Logie R. H. Stoltzfus E. R. Zacks R. T. (New York, NY: Oxford University Press), 66–88. 10.1093/acprof:oso/9780195100990.003.0003

[B39] Stroop J. R. (1935). Studies of interference in serial verbal reactions. J. Exp. Psychol. 18, 643–662. 10.1037/h0054651

[B40] Studebaker G. A. (1985). A rationalized arcsine transform. J. Speech Lang. Hear. Res. 28, 455–462. 10.1044/jshr.2803.455 4046587

[B41] Taler V. Aaron G. P. Steinmetz L. G. Pisoni D. B. (2010). Lexical neighborhood density effects on spoken word recognition and production in healthy aging. J. Gerontol. Psychol. Sci. 65B, 551–560. 10.1093/geronb/gbq039 PMC2920945 20542997

[B42] Van der Elst W. Van Boxtel M. P. Van Breukelen G. J. Jolles J. (2006). The Stroop color-word test: influence of age, sex, and education; and normative data for a large sample across the adult age range. Assessment 13, 62–79. 10.1177/1073191105283427 16443719

[B43] Van Dijk K. R. Van Gerven P. W. Van Boxtel M. P. Van der Elst W. Jolles J. (2008). No protective effects of education during normal cognitive aging: results from the 6-year follow-up of the Maastricht Aging Study. Psychol. Aging 23, 119. 10.1037/0882-7974.23.1.119 18361661

[B44] Zalonis I. Christidi F. Bonakis A. Kararizou E. Triantafyllou N. I. Paraskevas G.. (2009). The stroop effect in Greek healthy population: normative data for the Stroop Neuropsychological Screening Test. Arch. Clin. Neuropsychol. 24, 81–88. 10.1093/arclin/acp011 19395358

