# Supplementary material for: Visual Inhibition Measures Predict Speech-in-Noise Perception Only in People With Low Levels of Education
Source: Front Psychol. 2019 Jan 23;9:2779. doi: 10.3389/fpsyg.2018.02779 (PMC6357928; doi:10.3389/fpsyg.2018.02779)
Supplement: Supplementary file 1 [file Data_Sheet_1.pdf]

APPENDIX 1: MODEL SELECTION

A backwards stepwise procedure (Bolker et al, 2009; Matuschek et al, 2017) was used to determine the final set of predictors for each model. This Appendix contains details of the models run at each stage of the stepwise procedure. Effects removed at each stage are in italics. For brevity, we list only the dataset, the predictors (fixed effects) included and the results from significance testing (Type III ANOVA, Satterthwaite's method). Details of the final models can be found in the main text.

All models are linear mixed models (LMMs) using ML estimation, with predictor variables as fixed effects, Type 3 SS and participants as random intercepts.

Categorical fixed effects were: i) semantic predictability ("Pred"; LP/HP) of sentence-final words; ii) word frequency ("WF"; high/low) and iii) neighborhood density ("ND"; high/low) of isolated words; iv) speech type ("Type"; sentences/words); v) SNR ("SNR"; high/low).

Continuous predictors were: i) Stroop interference score ("SI"); ii) hearing sensitivity ("PTA"); iii) educational attainment ("Edu").

The specific backwards stepwise procedure was the same as that used in Knight & Heinrich (2017), as follows: the most complex possible model (i.e. full factorial: all main effects and all possible interactions) was run first. Non-significant effects (i.e. where p > 0.05) were then removed one level at a time. For example, if the highest-level interaction was a 4-way interaction and was not significant, it was removed and the model re-run. All non-significant 3-way interactions were then removed, and the model re-run. All non-significant 2-way interactions were then removed, and so on. If a significant higher-order interaction lost significance at any stage, this interaction was removed immediately before further changes were made. The principle of marginality was observed (for example, if A*B*C was kept in the model, then the model also included A*B, A*C and B*C). To achieve a balance between marginality and parsimoniousness, the following rules were observed: (1) Even if the highest-level interaction was significant, it was not included in the model if it contained 5 or more factors. This allowed the models to be reasonably trimmed in the first instance. (2) A lower-level significant 5- or 4-way interaction was only kept in the model if it contained the Stroop variable. (3) All significant and/or marginal 3-way and 2-way interactions were included, regardless of whether they contained the Stroop variable. (4) All main effects were kept in the model at all times.

In all cases, *** = < 0.001; ** = < 0.01; * = < 0.05.

A1.1 Combined dataset

**MODEL 1**		
**Fixed effects**	**F value (nomDF, denDF)**	**p value**
SI	1.94 (1, 46)	0.170
PTA	4.65 (1, 46)	0.036 *
Edu	5.58 (1, 46)	0.023 *
Type	11.32 (1, 138)	0.001 ***

SNR	8.52 (1, 138)	0.004 **
SI*PTA	0.57 (1, 46)	0.452
SI*Edu	7.53 (1, 46)	0.009 **
SI*Type	4.90 (1, 138)	0.029 *
SI*SNR	0.67 (1, 138)	0.413
PTA*Edu	1.32 (1, 46)	0.257
PTA*Type	6.59 (1, 138)	0.011 *
PTA*SNR	2.02 (1, 138)	0.158
Edu*Type	0.70 (1, 138)	0.403
Edu*SNR	0.59 (1, 138)	0.444
Type*SNR	0.19 (1, 138)	0.660
SI*PTA*Edu	0.67 (1, 46)	0.419
SI*PTA*Type	3.64 (1, 138)	0.058
SI*PTA*SNR	2.93 (1, 138)	0.089
SI*Edu*Type	0.82 (1, 138)	0.367
SI*Edu*SNR	0.40 (1, 138)	0.529
SI*Type*SNR	0.53 (1, 138)	0.469
PTA*Edu*Type	2.82 (1, 138)	0.096
PTA*Edu*SNR	0.48 (1, 138)	0.492
PTA*Type*SNR	0.60 (1, 138)	0.441
Edu*Type*SNR	0.03 (1, 138)	0.868
SI*PTA*Edu*Type	2.38 (1, 138)	0.125
SI*PTA*Edu*SNR	0.35 (1, 138)	0.557
SI*PTA*Type*SNR	1.23 (1, 138)	0.269
SI*Edu*Type*SNR	0.08 (1, 138)	0.776
PTA*Edu*Type*SNR	0.14 (1, 138)	0.708
*SI*PTA*Edu*Type*SNR*	*0.83 (1, 138)*	*0.365*

**MODEL 2**		

**Fixed effects**	**F value (nomDF, denDF)**	**p value**
SI	1.94 (1, 46)	0.170
PTA	4.65 (1, 46)	0.036 *
Edu	5.58 (1, 46)	0.023 *
Type	11.25 (1, 138)	0.001 **
SNR	8.47 (1, 138)	0.004 **
SI*PTA	0.57 (1, 46)	0.452
SI*Edu	7.53 (1, 46)	0.009 **
SI*Type	4.87 (1, 138)	0.029 *
SI*SNR	0.67 (1, 138)	0.415
PTA*Edu	1.32 (1, 46)	0.257
PTA*Type	6.55 (1, 138)	0.012 *
PTA*SNR	2.00 (1, 138)	0.159
Edu*Type	0.70 (1, 138)	0.404
Edu*SNR	0.59 (1, 138)	0.445
Type*SNR	1.27 (1, 138)	0.262
SI*PTA*Edu	0.67 (1, 46)	0.419
SI*PTA*Type	3.62 (1, 138)	0.059
SI*PTA*SNR	2.91 (1, 138)	0.090
SI*Edu*Type	0.81 (1, 138)	0.368
SI*Edu*SNR	0.40 (1, 138)	0.530
SI*Type*SNR	0.07 (1, 138)	0.798
PTA*Edu*Type	2.80 (1, 138)	0.097
PTA*Edu*SNR	0.47 (1, 138)	0.493
PTA*Type*SNR	0.81 (1, 138)	0.371
Edu*Type*SNR	0.39 (1, 138)	0.533
*SI*PTA*Edu*Type*	*2.37 (1, 138)*	*0.126*
*SI*PTA*Edu*SNR*	*0.35 (1, 138)*	*0.558*
*SI*PTA*Type*SNR*	*1.60 (1, 138)*	*0.208*

*SI*Edu*Type*SNR*	*0.50 (1, 138)*	*0.482*
*PTA*Edu*Type*SNR*	*3.65 (1, 138)*	*0.058*

**MODEL 3**		
**Fixed effects**	**F value (nomDF, denDF)**	**p value**
SI	1.94 (1, 46)	0.170
PTA	4.65 (1, 46)	0.036 *
Edu	5.58 (1, 46)	0.023 *
Type	8.55 (1, 138)	0.004 **
SNR	14.25 (1, 138)	< 0.001 ***
SI*PTA	0.57 (1, 46)	0.452
SI*Edu	7.53 (1, 46)	0.009 **
SI*Type	2.49 (1, 138)	0.117
SI*SNR	0.33 (1, 138)	0.567
PTA*Edu	1.32 (1, 46)	0.257
PTA*Type	7.36 (1, 138)	0.008 **
PTA*SNR	2.15 (1, 138)	0.145
Edu*Type	2.67 (1, 138)	0.105
Edu*SNR	1.23 (1, 138)	0.270
Type*SNR	1.06 (1, 138)	0.306
*SI*PTA*Edu*	*0.67 (1, 46)*	*0.419*
SI*PTA*Type	4.47 (1, 138)	0.036 *
*SI*PTA*SNR*	*3.14 (1, 138)*	*0.079*
*SI*Edu*Type*	*2.60 (1, 138)*	*0.109*
*SI*Edu*SNR*	*0.84 (1, 138)*	*0.360*
*SI*Type*SNR*	*0.01 (1, 138)*	*0.935*
*PTA*Edu*Type*	*0.49 (1, 138)*	*0.485*
*PTA*Edu*SNR*	*0.20 (1, 138)*	*0.655*
*PTA*Type*SNR*	*0.11 (1, 138)*	*0.738*

*Edu*Type*SNR*	*0.04 (1, 138)*	*0.842*

**MODEL 4**		
**Fixed effects**	**F value (nomDF, denDF)**	**p value**
SI	1.26 (1, 46)	0.268
PTA	4.21 (1, 46)	0.046 *
Edu	4.90 (1, 46)	0.032 *
Type	12.39 (1, 138)	0.001 ***
SNR	11.85 (1, 138)	0.001 ***
SI*PTA	0.40 (1, 46)	0.530
SI*Edu	6.85 (1, 46)	0.012 *
SI*Type	4.57 (1, 138)	0.034 *
SI*SNR	0.94 (1, 138)	0.334
PTA*Edu	1.87 (1, 46)	0.178
PTA*Type	4.31 (1, 138)	0.040 *
PTA*SNR	1.09 (1, 138)	0.299
Edu*Type	0.21 (1, 138)	0.651
Edu*SNR	1.36 (1, 138)	0.246
Type*SNR	15.72 (1, 138)	< 0.001 ***
*SI*PTA*Type*	*2.15 (1, 138)*	*0.145*

**MODEL 5**		
**Fixed effects**	**F value (nomDF, denDF)**	**p value**
SI	1.26 (1, 46)	0.268
PTA	4.21 (1, 46)	0.046 *
Edu	4.90 (1, 46)	0.032 *
Type	13.00 (1, 138)	< 0.001 ***
SNR	11.66 (1, 138)	0.001 ***
*SI*PTA*	*0.40 (1, 46)*	*0.530*

SI*Edu	6.85 (1, 46)	0.012 *
SI*Type	4.81 (1, 138)	0.030 *
*SI*SNR*	*0.92 (1, 138)*	*0.338*
*PTA*Edu*	*1.87 (1, 46)*	*0.178*
PTA*Type	4.74 (1, 138)	0.031 *
*PTA*SNR*	*1.07 (1, 138)*	*0.303*
*Edu*Type*	*0.60 (1, 138)*	*0.441*
*Edu*SNR*	*1.34 (1, 138)*	*0.250*
Type*SNR	15.48 (1, 138)	< 0.001 ***

A1.2 Sentence task

**MODEL 1**		
**Fixed effects**	**F value (nomDF, denDF)**	**p value**
SI	0.23 (1, 46)	0.637
PTA	8.94 (1, 46)	0.004 **
Edu	6.92 (1, 46)	0.012 *
Pred	45.03 (1, 138)	< 0.001 ***
SNR	3.41 (1, 138)	0.067
SI*PTA	1.89 (1, 46)	0.176
SI*Edu	9.16 (1, 46)	0.004 **
SI*Pred	0.98 (1, 138)	0.324
SI*SNR	1.48 (1, 138)	0.226
PTA*Edu	0.35 (1, 46)	0.557
PTA*Pred	0.51 (1, 138)	0.474
PTA*SNR	1.64 (1, 138)	0.202
Edu*Pred	0.09 (1, 138)	0.760
Edu*SNR	0.19 (1, 138)	0.667
Pred*SNR	6.17 (1, 138)	0.014 *
SI*PTA*Edu	0.11 (1, 46)	0.736

SI*PTA*Pred	1.61 (1, 138)	0.207
SI*PTA*SNR	2.84 (1, 138)	0.094
SI*Edu*Pred	0.04 (1, 138)	0.844
SI*Edu*SNR	0.16 (1, 138)	0.691
SI*Pred*SNR	8.18 (1, 138)	0.005 **
PTA*Edu*Pred	0.11 (1, 138)	0.739
PTA*Edu*SNR	1.08 (1, 138)	0.301
PTA*Pred*SNR	0.02 (1, 138)	0.894
Edu*Pred*SNR	3.47 (1, 138)	0.065
SI*PTA*Edu*Pred	0.01 (1, 138)	0.926
SI*PTA*Edu*SNR	1.99 (1, 138)	0.160
SI*PTA*Pred*SNR	0.34 (1, 138)	0.562
SI*Edu*Pred*SNR	3.26 (1, 138)	0.073
PTA*Edu*Pred*SNR	2.72 (1, 138)	0.102
*SI*PTA*Edu*Pred*SNR*	*4.60 (1, 138)*	*0.034 **

**MODEL 2**		
**Fixed effects**	**F value (nomDF, denDF)**	**p value**
SI	0.23 (1, 46)	0.637
PTA	8.94 (1, 46)	0.004 **
Edu	6.92 (1, 46)	0.012 *
Pred	43.57 (1, 138)	< 0.001 ***
SNR	3.30 (1, 138)	0.071
SI*PTA	1.89 (1, 46)	0.176
SI*Edu	9.16 (1, 46)	0.004 **
SI*Pred	0.95 (1, 138)	0.332
SI*SNR	1.43 (1, 138)	0.234
PTA*Edu	0.35 (1, 46)	0.557
PTA*Pred	0.50 (1, 138)	0.481

PTA*SNR	1.59 (1, 138)	0.210
Edu*Pred	0.09 (1, 138)	0.764
Edu*SNR	0.18 (1, 138)	0.672
Pred*SNR	2.35 (1, 138)	0.127
SI*PTA*Edu	0.11 (1, 46)	0.736
SI*PTA*Pred	1.56 (1, 138)	0.214
SI*PTA*SNR	2.75 (1, 138)	0.100
SI*Edu*Pred	0.04 (1, 138)	0.847
SI*Edu*SNR	0.15 (1, 138)	0.696
SI*Pred*SNR	3.86 (1, 138)	0.051
PTA*Edu*Pred	0.11 (1, 138)	0.743
PTA*Edu*SNR	1.04 (1, 138)	0.309
PTA*Pred*SNR	0.17 (1, 138)	0.681
Edu*Pred*SNR	1.02 (1, 138)	0.313
*SI*PTA*Edu*Pred*	*0.01 (1, 138)*	*0.927*
*SI*PTA*Edu*SNR*	*1.93 (1, 138)*	*0.167*
*SI*PTA*Pred*SNR*	*0.84 (1, 138)*	*0.362*
*SI*Edu*Pred*SNR*	*1.04 (1, 138)*	*0.311*
*PTA*Edu*Pred*SNR*	*2.50 (1, 138)*	*0.116*

**MODEL 3**		
**Fixed effects**	**F value (nomDF, denDF)**	**p value**
SI	0.23 (1, 46)	0.637
PTA	8.95 (1, 46)	0.004 **
Edu	6.92 (1, 46)	0.012 *
Pred	59.80 (1, 138)	< 0.001 ***
SNR	9.03 (1, 138)	0.003 **
SI*PTA	1.89 (1, 46)	0.176
SI*Edu	9.16 (1, 46)	0.004 **

SI*Pred	1.45 (1, 138)	0.231
SI*SNR	0.23 (1, 138)	0.629
PTA*Edu	0.35 (1, 46)	0.557
PTA*Pred	0.46 (1, 138)	0.499
PTA*SNR	1.99 (1, 138)	0.160
Edu*Pred	0.14 (1, 138)	0.713
Edu*SNR	1.24 (1, 138)	0.268
Pred*SNR	1.54 (1, 138)	0.217
*SI*PTA*Edu*	*0.11 (1, 46)*	*0.736*
*SI*PTA*Pred*	*1.47 (1, 138)*	*0.228*
*SI*PTA*SNR*	*3.39 (1, 138)*	*0.068*
*SI*Edu*Pred*	*0.06 (1, 138)*	*0.808*
*SI*Edu*SNR*	*1.00 (1, 138)*	*0.318*
*SI*Pred*SNR*	*2.26 (1, 138)*	*0.135*
*PTA*Edu*Pred*	*0.80 (1, 138)*	*0.374*
*PTA*Edu*SNR*	*1.40 (1, 138)*	*0.240*
*PTA*Pred*SNR*	*1.54 (1, 138)*	*0.217*
*Edu*Pred*SNR*	*0.00 (1, 138)*	*0.964*

**MODEL 4**		
**Fixed effects**	**F value (nomDF, denDF)**	**p value**
SI	0.12 (1, 46)	0.733
PTA	8.81 (1, 46)	0.005 **
Edu	7.60 (1, 46)	0.008 **
Pred	59.98 (1, 138)	< 0.001 ***
SNR	7.41 (1, 138)	0.007 **
*SI*PTA*	*1.78 (1, 46)*	*0.188*
SI*Edu	9.93 (1, 46)	0.003 **
*SI*Pred*	*1.08 (1, 138)*	*0.300*

*SI*SNR*	*0.30 (1, 138)*	*0.583*
*PTA*Edu*	*1.02 (1, 46)*	*0.318*
*PTA*Pred*	*2.18 (1, 138)*	*0.142*
*PTA*SNR*	*0.60 (1, 138)*	*0.439*
*Edu*Pred*	*0.06 (1, 138)*	*0.802*
*Edu*SNR*	*0.46 (1, 138)*	*0.500*
*Pred*SNR*	*0.54 (1, 138)*	*0.462*

A1.3 Word task

**MODEL 1**		
**Fixed effects**	**F value (nomDF, denDF)**	**p value**
SI	3.67 (1, 46)	0.062
PTA	1.06 (1, 46)	0.310
Edu	3.23 (1, 46)	0.079
SNR	5.48 (1, 322)	0.020 *
WF	2.98 (1, 322)	0.085
ND	9.05 (1, 322)	0.003 **
SI*PTA	0.00 (1, 46)	0.967
SI*Edu	4.45 (1, 46)	0.040 *
SI*SNR	0.00 (1, 322)	0.997
SI*WF	0.03 (1, 322)	0.854
SI*ND	4.29 (1, 322)	0.039 *
PTA*Edu	2.39 (1, 46)	0.129
PTA*SNR	0.52 (1, 322)	0.473
PTA*WF	0.02 (1, 322)	0.897
PTA*ND	5.17 (1, 322)	0.024 *
Edu*SNR	0.36 (1, 322)	0.547
Edu*WF	2.61 (1, 322)	0.107
Edu*ND	0.81 (1, 322)	0.368

SNR*WF	1.77 (1, 322)	0.184
SNR*ND	0.00 (1, 322)	0.994
WF*ND	2.65 (1, 322)	0.104
SI*PTA*Edu	1.51 (1, 46)	0.225
SI*PTA*SNR	0.41 (1, 322)	0.520
SI*PTA*WF	0.49 (1, 322)	0.485
SI*PTA*ND	5.23 (1, 322)	0.023 *
SI*Edu*SNR	0.17 (1, 322)	0.684
SI*Edu*WF	2.30 (1, 322)	0.131
SI*Edu*ND	2.41 (1, 322)	0.122
SI*SNR*WF	0.71 (1, 322)	0.399
SI*SNR*ND	0.03 (1, 322)	0.864
SI*WF*ND	1.57 (1, 322)	0.211
PTA*Edu*SNR	0.07 (1, 322)	0.787
PTA*Edu*WF	1.80 (1, 322)	0.180
PTA*Edu*ND	0.02 (1, 322)	0.890
PTA*SNR*WF	0.78 (1, 322)	0.377
PTA*SNR*ND	2.08 (1, 322)	0.150
PTA*WF*ND	0.09 (1, 322)	0.770
Edu*SNR*WF	0.13 (1, 322)	0.723
Edu*SNR*ND	0.45 (1, 322)	0.502
Edu*WF*ND	0.16 (1, 322)	0.691
SNR*WF*ND	0.00 (1, 322)	0.978
SI*PTA*Edu*SNR	0.11 (1, 322)	0.740
SI*PTA*Edu*WF	1.60 (1, 322)	0.207
SI*PTA*Edu*ND	0.08 (1, 322)	0.784
SI*PTA*SNR*WF	0.63 (1, 322)	0.428
SI*PTA*SNR*ND	2.74 (1, 322)	0.100
SI*PTA*WF*ND	0.22 (1, 322)	0.636

SI*Edu*SNR*WF	0.01 (1, 322)	0.943
SI*Edu*SNR*ND	0.46 (1, 322)	0.500
SI*Edu*WF*ND	0.00 (1, 322)	0.954
SI*SNR*WF*ND	0.01 (1, 322)	0.933
PTA*Edu*SNR*WF	0.45 (1, 322)	0.505
PTA*Edu*SNR*ND	0.63 (1, 322)	0.430
PTA*Edu*WF*ND	1.66 (1, 322)	0.199
PTA*SNR*WF*ND	1.11 (1, 322)	0.292
Edu*SNR*WF*ND	0.09 (1, 322)	0.770
SI*PTA*Edu*SNR*WF	0.36 (1, 322)	0.551
SI*PTA*Edu*SNR*ND	0.28 (1, 322)	0.599
SI*PTA*Edu*WF*ND	1.54 (1, 322)	0.216
SI*PTA*SNR*WF*ND	0.78 (1, 322)	0.377
SI*Edu*SNR*WF*ND	0.03 (1, 322)	0.857
PTA*Edu*SNR*WF*ND	0.51 (1, 322)	0.475
*SI*PTA*Edu*SNR*WF*ND*	*0.15 (1, 322)*	*0.698*

**MODEL 2**		
**Fixed effects**	**F (nomDF, denDF)**	**p value**
SI	3.67 (1, 46)	0.062
PTA	1.06 (1, 46)	0.310
Edu	3.23 (1, 46)	0.079
SNR	5.48 (1, 322)	0.020 *
WF	2.97 (1, 322)	0.086
ND	9.05 (1, 322)	0.003 **
SI*PTA	0.00 (1, 46)	0.967
SI*Edu	4.45 (1, 46)	0.040 *
SI*SNR	0.00 (1, 322)	0.997
SI*WF	0.03 (1, 322)	0.854

SI*ND	4.29 (1, 322)	0.039 *
PTA*Edu	2.39 (1, 46)	0.129
PTA*SNR	0.52 (1, 322)	0.473
PTA*WF	0.02 (1, 322)	0.897
PTA*ND	5.17 (1, 322)	0.024 *
Edu*SNR	0.36 (1, 322)	0.547
Edu*WF	2.61 (1, 322)	0.107
Edu*ND	0.81 (1, 322)	0.368
SNR*WF	1.77 (1, 322)	0.184
SNR*ND	0.00 (1, 322)	0.994
WF*ND	2.65 (1, 322)	0.104
SI*PTA*Edu	1.51 (1, 46)	0.225
SI*PTA*SNR	0.41 (1, 322)	0.520
SI*PTA*WF	0.49 (1, 322)	0.485
SI*PTA*ND	5.23 (1, 322)	0.023 *
SI*Edu*SNR	0.17 (1, 322)	0.684
SI*Edu*WF	2.29 (1, 322)	0.131
SI*Edu*ND	2.41 (1, 322)	0.122
SI*SNR*WF	0.71 (1, 322)	0.399
SI*SNR*ND	0.03 (1, 322)	0.864
SI*WF*ND	1.57 (1, 322)	0.211
PTA*Edu*SNR	0.07 (1, 322)	0.787
PTA*Edu*WF	1.80 (1, 322)	0.180
PTA*Edu*ND	0.02 (1, 322)	0.890
PTA*SNR*WF	0.78 (1, 322)	0.377
PTA*SNR*ND	2.08 (1, 322)	0.150
PTA*WF*ND	0.09 (1, 322)	0.770
Edu*SNR*WF	0.13 (1, 322)	0.723
Edu*SNR*ND	0.45 (1, 322)	0.502

Edu*WF*ND	0.16 (1, 322)	0.691
SNR*WF*ND	0.05 (1, 322)	0.823
SI*PTA*Edu*SNR	0.11 (1, 322)	0.740
SI*PTA*Edu*WF	1.60 (1, 322)	0.207
SI*PTA*Edu*ND	0.08 (1, 322)	0.784
SI*PTA*SNR*WF	0.63 (1, 322)	0.428
SI*PTA*SNR*ND	2.74 (1, 322)	0.099
SI*PTA*WF*ND	0.22 (1, 322)	0.636
SI*Edu*SNR*WF	0.01 (1, 322)	0.943
SI*Edu*SNR*ND	0.45 (1, 322)	0.501
SI*Edu*WF*ND	0.00 (1, 322)	0.954
SI*SNR*WF*ND	0.14 (1, 322)	0.713
PTA*Edu*SNR*WF	0.45 (1, 322)	0.505
PTA*Edu*SNR*ND	0.62 (1, 322)	0.430
PTA*Edu*WF*ND	1.66 (1, 322)	0.199
PTA*SNR*WF*ND	1.24 (1, 322)	0.266
Edu*SNR*WF*ND	0.02 (1, 322)	0.892
*SI*PTA*Edu*SNR*WF*	*0.36 (1, 322)*	*0.551*
*SI*PTA*Edu*SNR*ND*	*0.28 (1, 322)*	*0.599*
*SI*PTA*Edu*WF*ND*	*1.54 (1, 322)*	*0.216*
*SI*PTA*SNR*WF*ND*	*0.92 (1, 322)*	*0.339*
*SI*Edu*SNR*WF*ND*	*0.13 (1, 322)*	*0.715*
*PTA*Edu*SNR*WF*ND*	*1.70 (1, 322)*	*0.193*

**MODEL 3**		
**Fixed effects**	**F value (nomDF, denDF)**	**p value**
SI	3.67 (1, 46)	0.062
PTA	1.06 (1, 46)	0.310
Edu	3.23 (1, 46)	0.079

SNR	5.39 (1, 322)	0.021 *
WF	2.93 (1, 322)	0.088
ND	8.91 (1, 322)	0.003 **
SI*PTA	0.00 (1, 46)	0.967
SI*Edu	4.45 (1, 46)	0.040 *
SI*SNR	0.00 (1, 322)	0.997
SI*WF	0.03 (1, 322)	0.855
SI*ND	4.22 (1, 322)	0.041 *
PTA*Edu	2.39 (1, 46)	0.129
PTA*SNR	0.51 (1, 322)	0.476
PTA*WF	0.02 (1, 322)	0.898
PTA*ND	5.09 (1, 322)	0.025 *
Edu*SNR	0.36 (1, 322)	0.551
Edu*WF	2.57 (1, 322)	0.110
Edu*ND	0.80 (1, 322)	0.372
SNR*WF	1.42 (1, 322)	0.234
SNR*ND	0.11 (1, 322)	0.737
WF*ND	7.57 (1, 322)	0.006 **
SI*PTA*Edu	1.51 (1, 46)	0.225
SI*PTA*SNR	0.41 (1, 322)	0.524
SI*PTA*WF	0.48 (1, 322)	0.488
SI*PTA*ND	5.15 (1, 322)	0.024 *
SI*Edu*SNR	0.16 (1, 322)	0.687
SI*Edu*WF	2.26 (1, 322)	0.134
SI*Edu*ND	2.37 (1, 322)	0.125
SI*SNR*WF	0.37 (1, 322)	0.542
SI*SNR*ND	0.02 (1, 322)	0.880
SI*WF*ND	0.44 (1, 322)	0.507
PTA*Edu*SNR	0.07 (1, 322)	0.789

PTA*Edu*WF	1.78 (1, 322)	0.184
PTA*Edu*ND	0.02 (1, 322)	0.891
PTA*SNR*WF	0.65 (1, 322)	0.420
PTA*SNR*ND	1.89 (1, 322)	0.170
PTA*WF*ND	0.02 (1, 322)	0.897
Edu*SNR*WF	0.46 (1, 322)	0.498
Edu*SNR*ND	0.99 (1, 322)	0.320
Edu*WF*ND	0.03 (1, 322)	0.873
SNR*WF*ND	0.00 (1, 322)	0.957
*SI*PTA*Edu*SNR*	*0.11 (1, 322)*	*0.742*
*SI*PTA*Edu*WF*	*1.57 (1, 322)*	*0.211*
*SI*PTA*Edu*ND*	*0.07 (1, 322)*	*0.786*
*SI*PTA*SNR*WF*	*0.49 (1, 322)*	*0.483*
*SI*PTA*SNR*ND*	*2.50 (1, 322)*	*0.115*
*SI*PTA*WF*ND*	*0.08 (1, 322)*	*0.780*
*SI*Edu*SNR*WF*	*0.03 (1, 322)*	*0.857*
*SI*Edu*SNR*ND*	*0.92 (1, 322)*	*0.339*
*SI*Edu*WF*ND*	*0.36 (1, 322)*	*0.549*
*SI*SNR*WF*ND*	*0.00 (1, 322)*	*0.979*
*PTA*Edu*SNR*WF*	*0.12 (1, 322)*	*0.730*
*PTA*Edu*SNR*ND*	*1.15 (1, 322)*	*0.285*
*PTA*Edu*WF*ND*	*0.12 (1, 322)*	*0.731*
*PTA*SNR*WF*ND*	*0.02 (1, 322)*	*0.898*
*Edu*SNR*WF*ND*	*1.47 (1, 322)*	*0.227*

**MODEL 4**		
**Fixed effects**	**F value (nomDF, denDF)**	**p value**
SI	3.67 (1, 46)	0.062
PTA	1.06 (1, 46)	0.310

Edu	3.23 (1, 46)	0.079
SNR	6.32 (1, 322)	0.012 *
WF	1.43 (1, 322)	0.232
ND	11.07 (1, 322)	0.001 ***
SI*PTA	0.00 (1, 46)	0.967
SI*Edu	4.45 (1, 46)	0.040 *
SI*SNR	0.05 (1, 322)	0.822
SI*WF	1.12 (1, 322)	0.292
SI*ND	5.09 (1, 322)	0.025 *
PTA*Edu	2.39 (1, 46)	0.129
PTA*SNR	0.44 (1, 322)	0.509
PTA*WF	0.00 (1, 322)	0.971
PTA*ND	5.12 (1, 322)	0.024 *
Edu*SNR	0.24 (1, 322)	0.622
Edu*WF	1.31 (1, 322)	0.252
Edu*ND	1.22 (1, 322)	0.271
SNR*WF	1.54 (1, 322)	0.215
SNR*ND	0.02 (1, 322)	0.890
WF*ND	7.09 (1, 322)	0.008 **
*SI*PTA*Edu*	*1.51 (1, 46)*	*0.225*
*SI*PTA*SNR*	*0.34 (1, 322)*	*0.561*
*SI*PTA*WF*	*0.24 (1, 322)*	*0.623*
SI*PTA*ND	5.25 (1, 322)	0.023 *
*SI*Edu*SNR*	*0.08 (1, 322)*	*0.771*
*SI*Edu*WF*	*1.15 (1, 322)*	*0.285*
*SI*Edu*ND*	*3.10 (1, 322)*	*0.079*
*SI*SNR*WF*	*0.49 (1, 322)*	*0.486*
*SI*SNR*ND*	*0.03 (1, 322)*	*0.854*
*SI*WF*ND*	*0.74 (1, 322)*	*0.389*

PTA*Edu*SNR	4.80 (1, 322)	0.029 *
*PTA*Edu*WF*	*0.21 (1, 322)*	*0.646*
*PTA*Edu*ND*	*2.24 (1, 322)*	*0.136*
*PTA*SNR*WF*	*0.27 (1, 322)*	*0.602*
*PTA*SNR*ND*	*0.13 (1, 322)*	*0.721*
*PTA*WF*ND*	*0.23 (1, 322)*	*0.635*
*Edu*SNR*WF*	*2.31 (1, 322)*	*0.129*
*Edu*SNR*ND*	*0.00 (1, 322)*	*0.957*
*Edu*WF*ND*	*2.04 (1, 322)*	*0.154*
*SNR*WF*ND*	*0.06 (1, 322)*	*0.809*

**MODEL 5**		
**Fixed effects**	**F value (nomDF, denDF)**	**p value**
SI	2.12 (1, 46)	0.152
PTA	0.74 (1, 46)	0.395
Edu	1.90 (1, 46)	0.175
SNR	5.99 (1, 322)	0.015 *
WF	0.90 (1, 322)	0.342
ND	14.44 (1, 322)	<0.001 ***
SI*PTA	0.02 (1, 46)	0.881
SI*Edu	3.02 (1, 46)	0.089
SI*SNR	0.09 (1, 322)	0.759
SI*WF	1.53 (1, 322)	0.217
SI*ND	5.82 (1, 322)	0.016 *
PTA*Edu	1.83 (1, 46)	0.183
PTA*SNR	0.12 (1, 322)	0.729
PTA*WF	2.24 (1, 322)	0.136
PTA*ND	3.09 (1, 322)	0.080
Edu*SNR	0.65 (1, 322)	0.421

Edu*WF	0.13 (1, 322)	0.720
Edu*ND	4.03 (1, 322)	0.046 *
SNR*WF	3.52 (1, 322)	0.062
SNR*ND	1.32 (1, 322)	0.252
WF*ND	156.04 (1, 322)	<0.001 ***
*SI*PTA*ND*	*2.41 (1, 322)*	*0.121*
PTA*Edu*SNR	4.89 (1, 322)	0.028 *

**MODEL 6**		
**Fixed effects**	**F value (nomDF, denDF)**	**p value**
SI	2.12 (1, 46)	0.152
PTA	0.74 (1, 46)	0.395
Edu	1.90 (1, 46)	0.175
SNR	5.95 (1, 322)	0.015 *
WF	0.9 (1, 322)	0.344
ND	15.26 (1, 322)	<0.001 ***
*SI*PTA*	*0.02 (1, 46)*	*0.881*
*SI*Edu*	*3.02 (1, 46)*	*0.089*
*SI*SNR*	*0.09 (1, 322)*	*0.760*
*SI*WF*	*1.52 (1, 322)*	*0.219*
SI*ND	6.16 (1, 322)	0.014 *
PTA*Edu	1.83 (1, 46)	0.183
PTA*SNR	0.12 (1, 322)	0.730
*PTA*WF*	*2.22 (1, 322)*	*0.137*
*PTA*ND*	*0.82 (1, 322)*	*0.365*
Edu*SNR	0.64 (1, 322)	0.423
*Edu*WF*	*0.13 (1, 322)*	*0.721*
*Edu*ND*	*2.94 (1, 322)*	*0.088*
*SNR*WF*	*3.49 (1, 322)*	*0.063*

Knight & Heinrich (2019)

*SNR*ND*	*1.31 (1, 322)*	*0.254*
WF*ND	154.88 (1, 322)	<0.001 ***
PTA*Edu*SNR	4.85 (1, 322)	0.028 *

References

Bolker, B. M., Brooks, M. E., Clark, C. J., Geange, S. W., Poulsen, J. R., Stevens, M. H. H., et al. (2009). Generalized linear mixed models: a practical guide for ecology and evolution. *Trends Ecol. Evol. 24*, 127–135. doi: 10.1016/j.tree.2008.10.008

Matuschek, H., Kliegl, R., Vasishth, S., Baayen, H., and Bates, D. (2017). Balancing Type I error and power in linear mixed models. *J. Mem. Lang. 94*, 305–315. doi: 10.1016/j.jml.2017.01.001
