# Supplementary material for: Visual Inhibition Measures Predict Speech-in-Noise Perception Only in People With Low Levels of Education
Source: Front Psychol. 2019 Jan 23;9:2779. doi: 10.3389/fpsyg.2018.02779 (PMC6357928; doi:10.3389/fpsyg.2018.02779)
Supplement: Supplementary file 2 [file Data_Sheet_2.pdf]

APPENDIX 2: CHECKING MODEL ASSUMPTIONS

A2.1 QQ plots of final model residuals

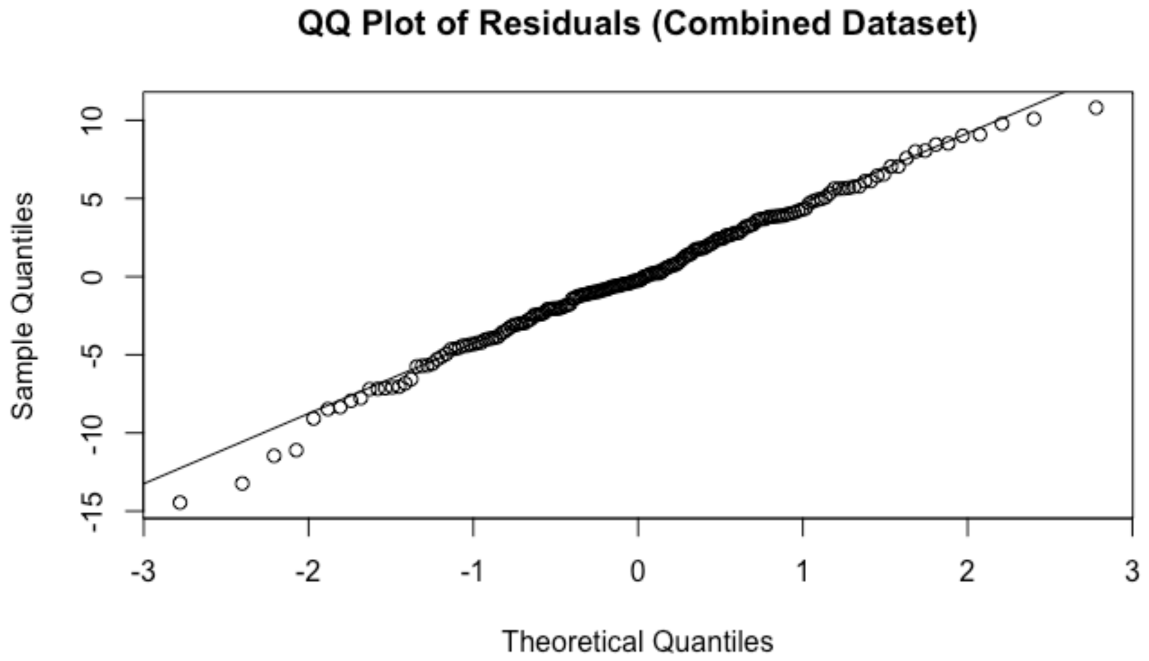

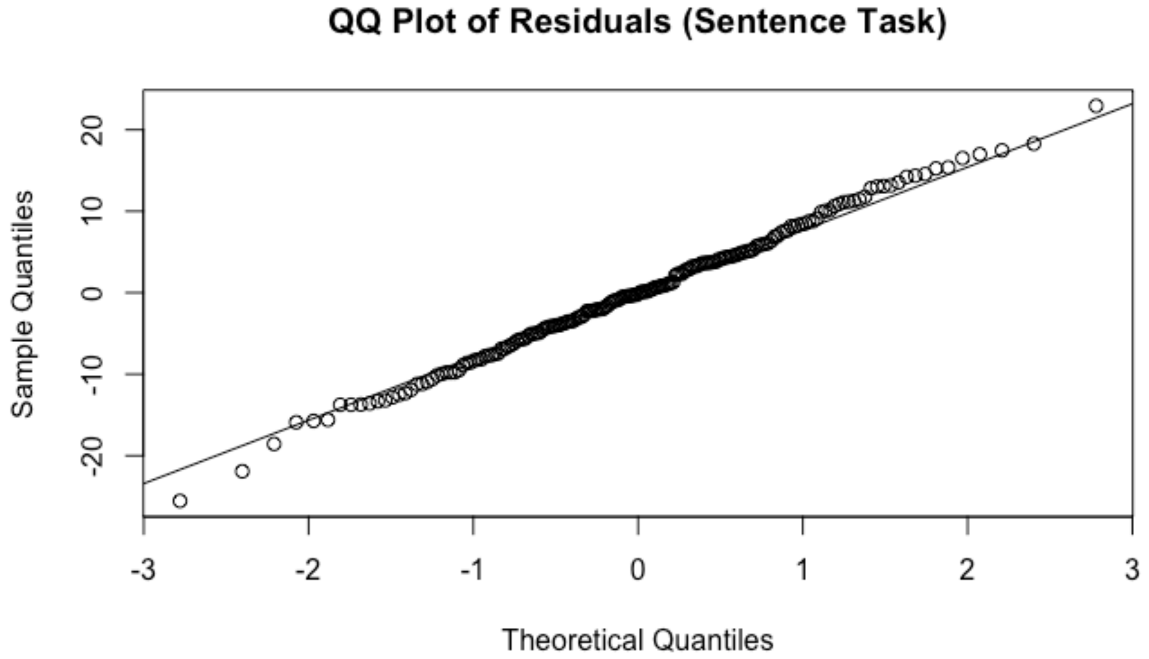

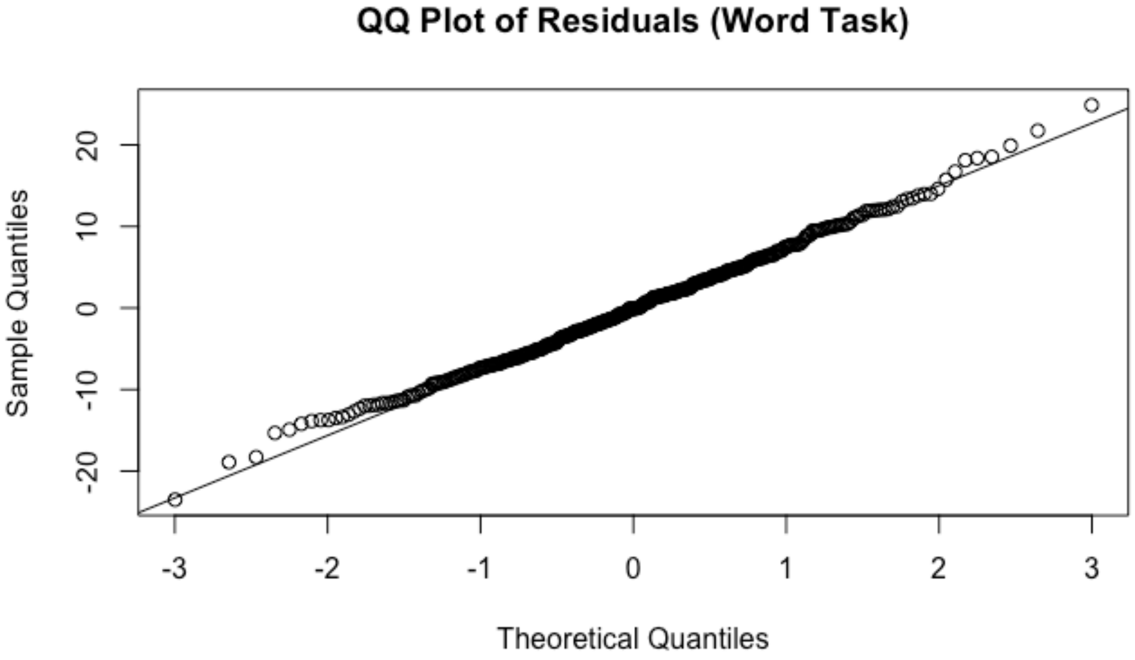

A2.2 QQ plots of final model random effects

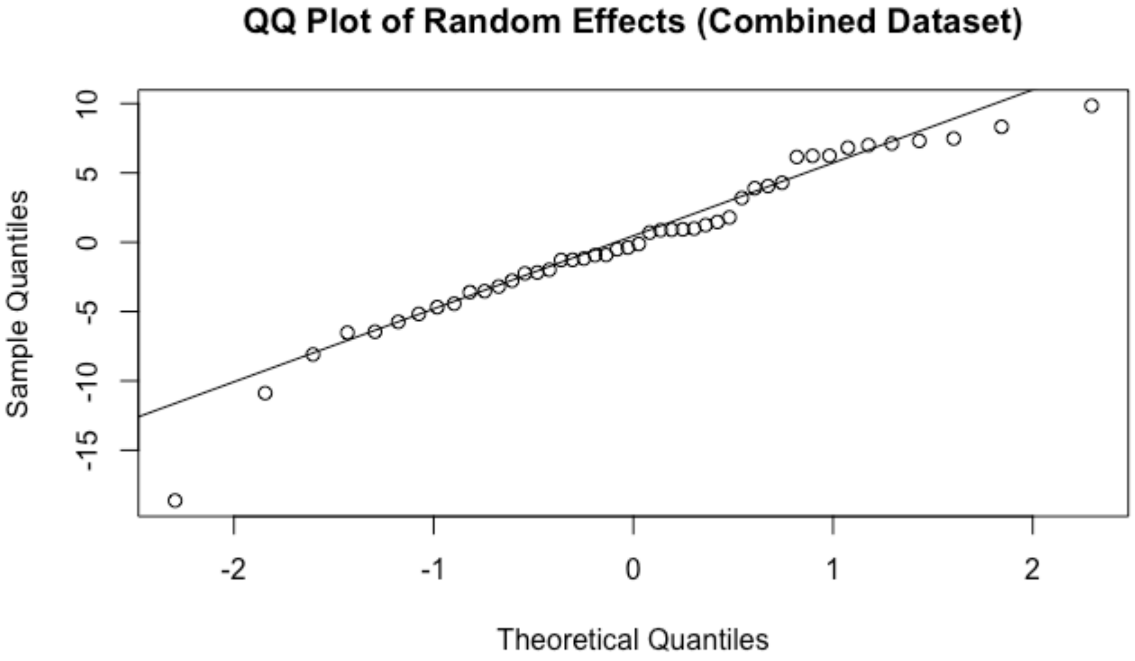

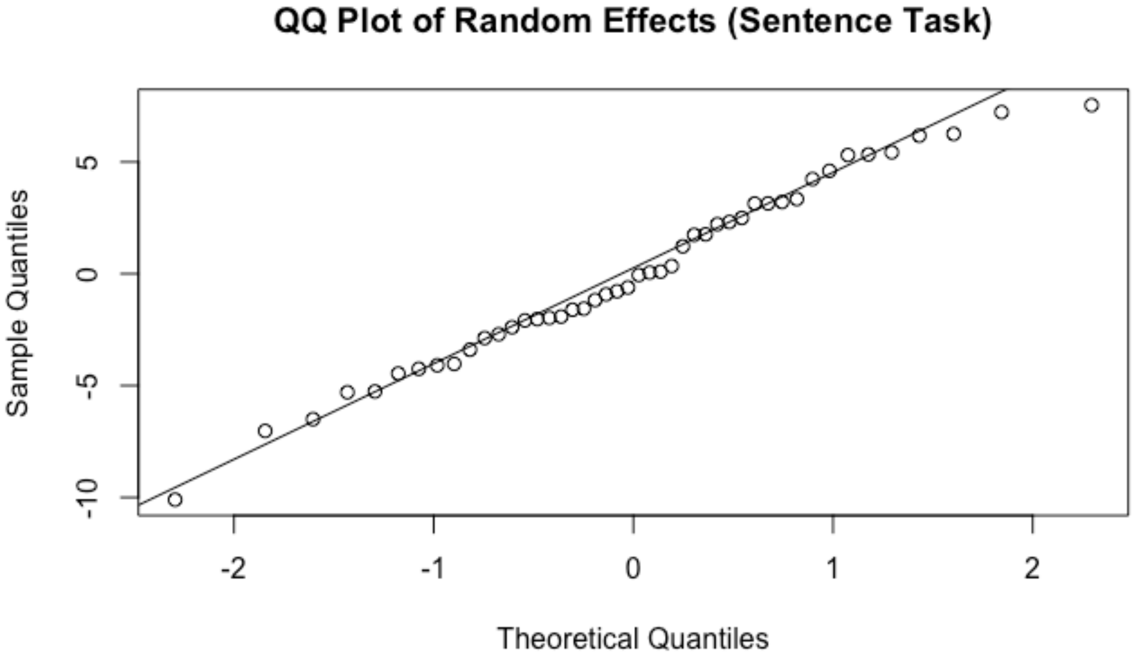
QQ Plot of Random Effects (Sentence Task)
Sample Quantiles
Theoretical Quantiles
5
0
-5
-10
-2
-1
0
1
2

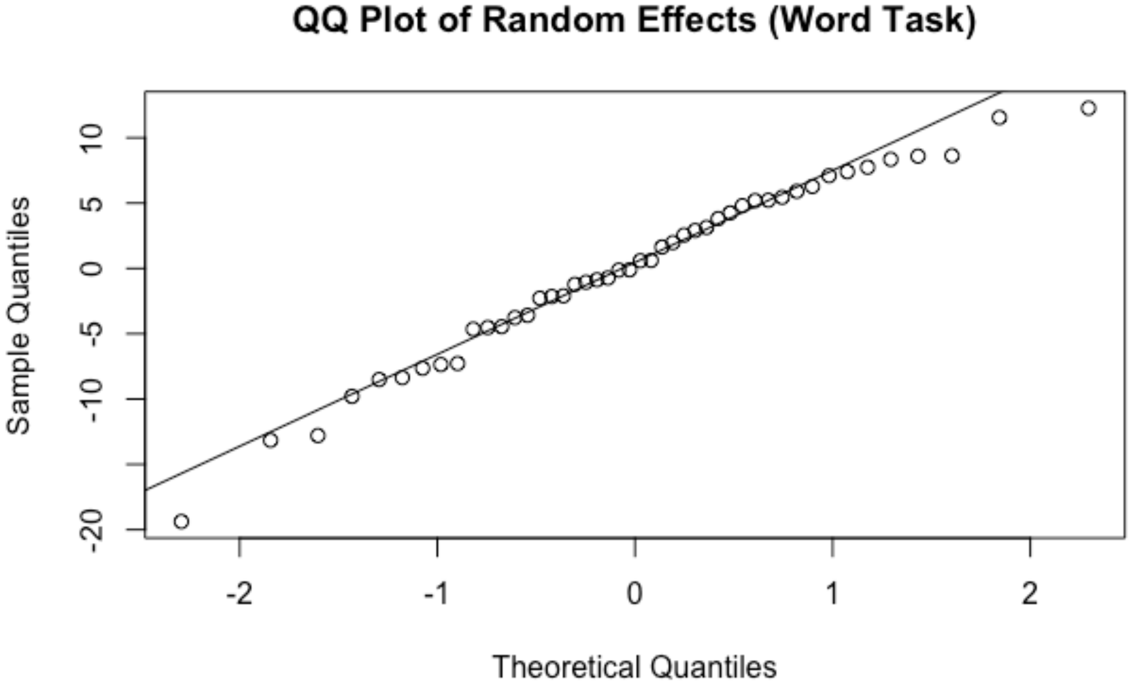
QQ Plot of Random Effects (Word Task)
Sample Quantiles
Theoretical Quantiles
10
5
0
-5
-10
-20
-2
-1
0
1
2

Knight & Heinrich (2019)

A2.3 Plots of final model predicted values vs. final model residuals

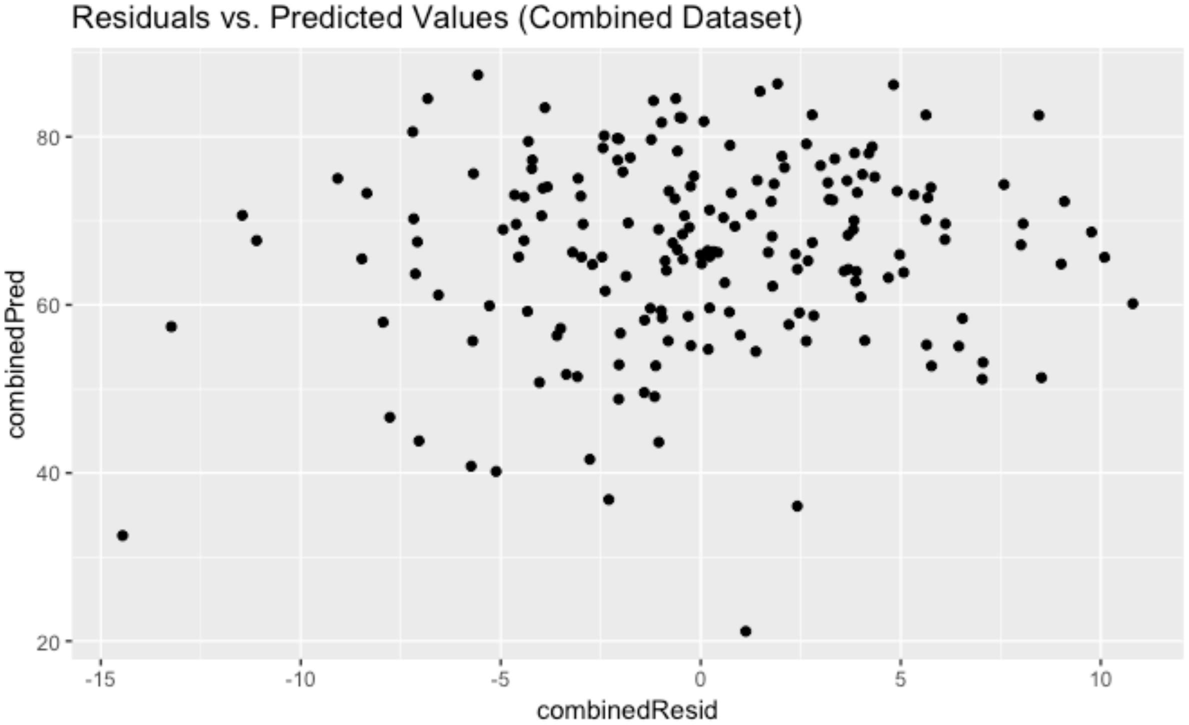

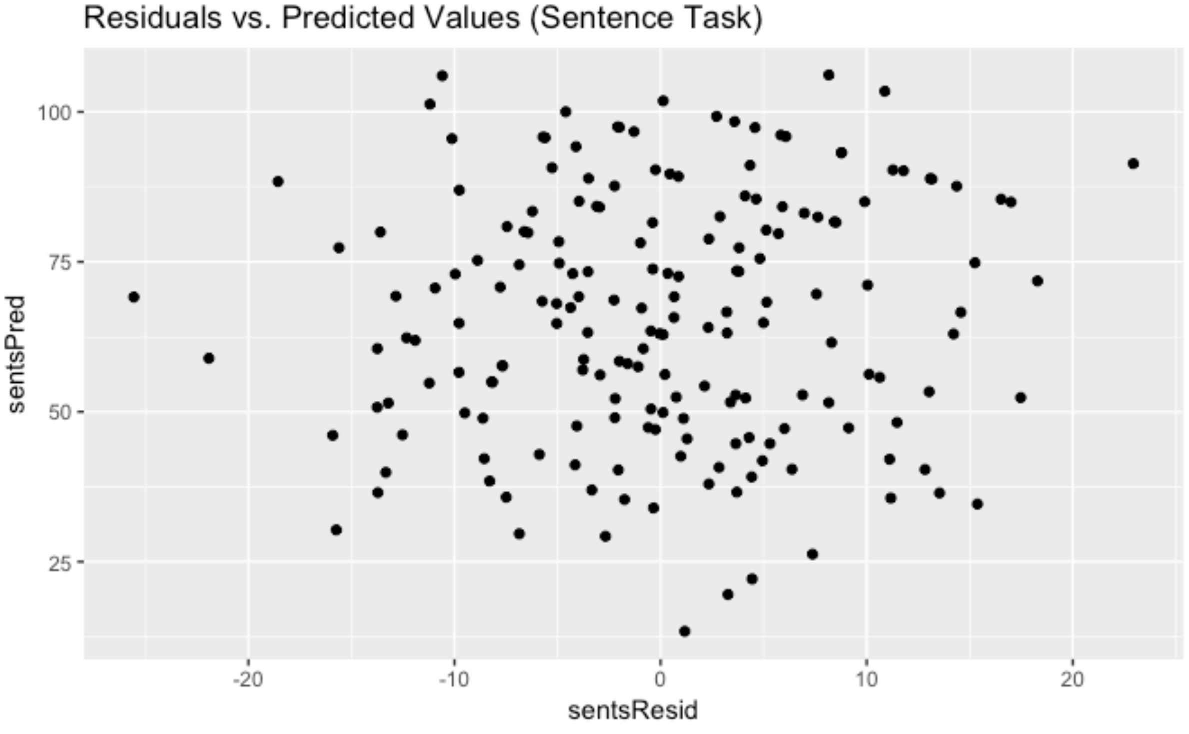

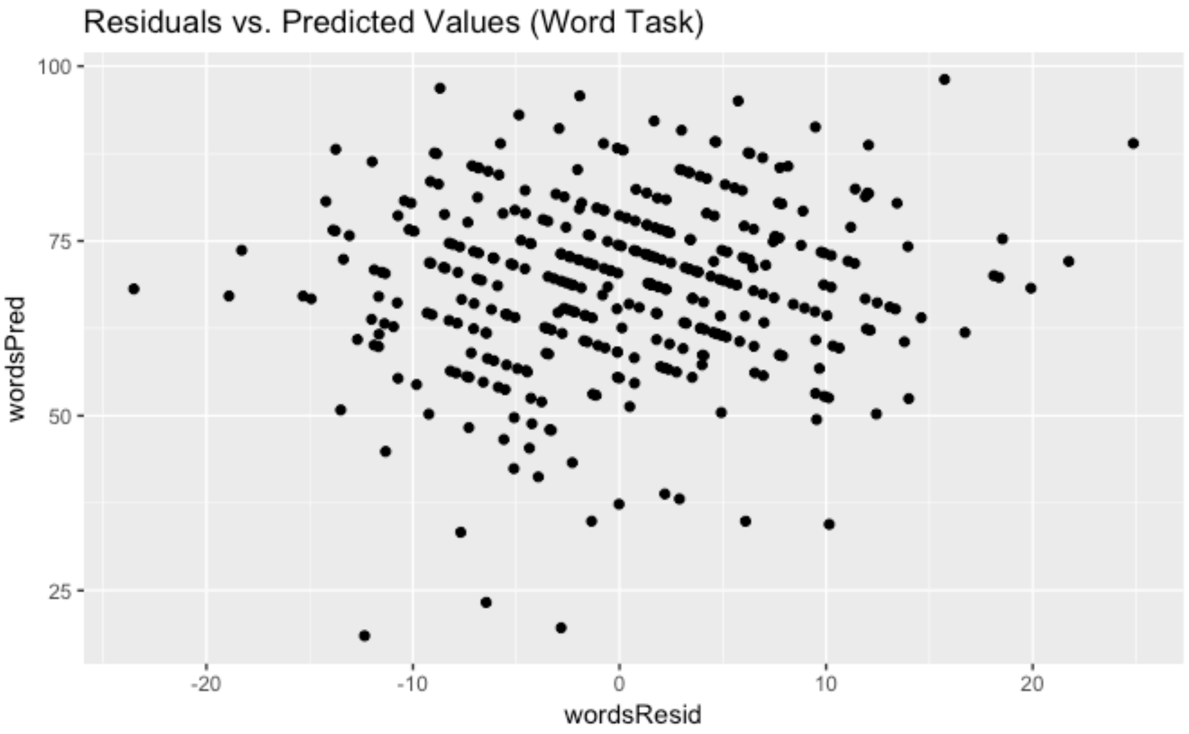
Residuals vs. Predicted Values (Word Task)
wordsPred
wordsResid
100
75
50
25
-20
-10
0
10
20
